# Dicer Regulates the Balance of Short-Lived Effector and Long-Lived Memory CD8 T Cell Lineages

**DOI:** 10.1371/journal.pone.0162674

**Published:** 2016-09-14

**Authors:** Florian M. Baumann, Yevgeniy Yuzefpolskiy, Surojit Sarkar, Vandana Kalia

**Affiliations:** 1 Department of Pediatrics, Division of Hematology and Oncology, University of Washington School of Medicine, Seattle, WA, United States of America; Ben Towne Center for Childhood Cancer Research, Seattle Children’s Research Institute, Seattle, WA, United States of America; 2 Department of Pathology, University of Washington School of Medicine, Seattle, WA, United States of America; 3 The Huck Institutes of Life Sciences, The Pennsylvania State University, University Park, PA, United States of America; Monash University, Australia, AUSTRALIA

## Abstract

MicroRNAs constitute a major post-transcriptional mechanism for controlling protein expression, and are emerging as key regulators during T cell development and function. Recent reports of augmented CD8 T cell activation and effector differentiation, and aberrant migratory properties upon ablation of Dicer/miRNAs in naïve cells have established a regulatory role of miRNAs during priming. Whether miRNAs continue to exert similar functions or are dispensable during later stages of CD8 T cell expansion and memory differentiation remains unclear. Here, we report a critical role of Dicer/miRNAs in regulating the balance of long-lived memory and short-lived terminal effector fates during the post-priming stages when CD8 T cells undergo clonal expansion to generate a large cytotoxic T lymphocyte (CTL) pool and subsequently differentiate into a quiescent memory state. Conditional ablation of Dicer/miRNAs in early effector CD8 T cells following optimal activation and expression of granzyme B, using unique *dicer*^*fl/fl*^ *gzmb-cre* mice, led to a strikingly diminished peak effector size relative to wild-type antigen-specific cells in the same infectious milieu. Diminished expansion of Dicer-ablated CD8 T cells was associated with lack of sustained antigen-driven proliferation and reduced accumulation of short-lived effector cells. Additionally, Dicer-ablated CD8 T cells exhibited more pronounced contraction after pathogen clearance and comprised a significantly smaller proportion of the memory pool, despite significantly higher proportions of CD127^Hi^ memory precursors at the effector peak. Combined with previous reports of dynamic changes in miRNA expression as CD8 T cells differentiate from naïve to effector and memory states, these findings support distinct stage-specific roles of miRNA-dependent gene regulation during CD8 T cell differentiation.

## Introduction

Effector and memory CD8 T cells play an important role in providing immunity against intracellular pathogens and in tumor control [1]. Effector CD8 T cells, or cytotoxic T lymphocytes (CTLs), possess immediate protective capacity by producing effector molecules such as granzyme B, perforin, IFN-γ and TNF-α, and by mounting cytotoxicity against infected or diseased target cells [2–4]. Memory CD8 T cells, on the other hand, mediate long-term protection by virtue of their ability to quiescently persist in the absence of antigen, and to elaborate potent effector responses immediately upon secondary infection or disease. Canonical memory cells typically arise after antigen clearance from a subset of effector CTLs [5], referred to as memory precursor effector cells (MPECs). MPECs express relatively higher levels of pro-survival molecules such as IL-7Rα and Bcl-2 than the short-lived effector cells (SLECs), and exhibit preferential survival, rapid downregulation of effector functions, and progressive acquisition of hallmark memory properties after antigen clearance. Consistent with dramatic differences in their phenotypic and functional states, effector and memory CD8 T cells express unique transcriptomic profiles [5–7]. However, the distinct gene regulatory mechanisms underlying the short-lived effector and long-lived memory lineages remain to be fully defined.

Recent microRNA profiling studies [8–10] have identified dynamic changes in the microRNA repertoire of naïve cells as they differentiate into effector and memory cells [8–10]. MicroRNAs, a class of short non-coding RNAs that are post-transcriptional inhibitors of gene expression, have emerged as major players in regulating the development and function of many immune cell-types [11]. With respect to T cells, miRNAs regulate thymic development of both CD4 and CD8 T cells [12] as well as the differentiation of mature T cells into various functional subsets. Consistent with largely suppressive functions ascribed to miRNAs, it has been shown that ablation of *dicer* in naïve CD8 T cells is associated with increased CD8 T cell activation, proliferation, and effector differentiation [13, 14]. However, in these studies aberrant activation and CD8 T cell localization associated with Dicer/miRNA loss prior to priming precluded analysis of memory differentiation.

To investigate the role of miRNAs in guiding short-lived effector and memory CTL differentiation after initial priming events, we employed a unique mouse model in which the RNase III enzyme Dicer (required for generation of most prototypical mature cellular miRNAs [15, 16]) is deleted specifically in early effector CD8 T cells after optimal stimulation. For this, we generated TCR transgenic mice with a *granzyme b-cre dicer*^*fl/fl*^ system. Granzyme B (GzmB) is a canonical effector molecule, whose expression is upregulated in all antigen-specific CD8 T cells after TCR stimulation [17][7][6]. Approaches of genetic tagging using *gzmb-cre* transgene have established that memory CD8 T cells, similar to SLECs, also pass through a GzmB+ effector phase [18][19][17]. Therefore, the *gzmb-cre dicer*^*fl/fl*^ system bypasses the requirement of Dicer during thymic development of T cells allowing investigation of miRNA regulation of effector and memory CD8 T cell differentiation events that ensue initial priming.

Ablation of miRNAs in a subset of effector CD8 T cells primed during acute Lymphocytic choriomeningitis virus (LCMV) infection led to a dramatic defect in expansion. Expansion defects were associated with loss of sustained proliferation and survival of SLECs, suggesting that miRNAs serve to drive SLEC responses during the post-priming stages. Interestingly, even though the MPEC numbers were unaffected at the effector peak in the absence of Dicer/miRNAs, the final memory numbers were severely compromised in antigen-specific CD8 T cells lacking Dicer. These studies reveal that the balance between effector and memory differentiation is heavily dependent on the regulatory functions of miRNAs in a CD8 T cell during priming [13, 14]. This study presents a distinct pro-proliferative, pro-survival role of miRNAs during post-activation stages of CD8 T cell responses to regulate the differentiation of terminal effector and memory lineages.

## Materials and Methods

### Ethics Statement

This study was carried out in strict accordance with the recommendations in the Guide for the Care and Use of Laboratory Animals of the National Institutes of Health. The Institutional Animal Care and Use Committee of the Pennsylvania State University and the Seattle Children’s Research Institute approved the protocols. All efforts were made to minimize suffering. All animals were euthanized using CO_2_ euthanasia followed by cervical dislocation based on endpoint criteria as approved by the university IACUC.

### Mice

C57BL/6 mice (Thy1.2^+^, Thy1.1^+^) were purchased from the Jackson Laboratory (Bar Harbor, ME). Thy1.1^+^ P14 mice bearing the D^b^GP33-specific T cell receptor (TCR) were fully backcrossed to C57BL/6 mice and were maintained in our animal colony. Floxed mice for conditional deletion of Dicer (B6.Cg-Dicer1^tm1Bdh^/J) were purchased from the Jackson Laboratory (stock number: 006366). In these mice, the exon encoding most of the RNase III catalytic domain of Dicer is flanked by LoxP sites. Cre recombinase-mediated excision of this exon results in functional inactivation of Dicer via deletion of 90 amino acids. The *dicer*^*fl/fl*^ mice were crossed with *gzmb-cre* mice [18] (a generous gift from Dr. J. Jacob, Emory University), in which Cre recombinase is expressed under control of the granzyme B promoter, to generate conditional knockout (CKO) mice. D^b^GP33-specific CD8 T cell Dicer^CKO^ mice were also generated by crossing Thy1.1^+^ P14 *gzmb-cre* mice bearing the D^b^GP33-specific T cell receptor with *dicer*^*fl/fl*^ mice.

### Virus and infections

The Armstrong strain of lymphocytic choriomeningitis virus (LCMV) was propagated, titered, and used as previously described [6, 7]. Mice were infected with 2×10^5^ LCMV intraperitoneally to initiate the infection.

### Antibodies, flow cytometry, and intracellular cytokine staining

All antibodies were purchased from Biolegend (San Diego, CA). Cells were stained for surface or intracellular proteins and cytokines as previously described [6, 7]. For analysis of intracellular cytokines, 10^6^ lymphocytes were stimulated with 0.2 μg/mL GP33-41 peptide in the presence of brefeldin A for 5 hours, followed by surface staining for CD8, Thy1.1, or Thy 1.2, and intracellular staining for IFN-γ, TNF-α, and IL-2. For caspase staining, the FAM-FLICA Apoptosis Detection Kit (Caspase 3 & 7 Assay Kit, green) was used (Neuromics, Minneapolis, MN). Cells were stained according to the manufacturer’s protocol in a 96-well plate, using 20 min incubation at 37°C together with CD8 PE-Cy7, Thy1.1 Alexa 700, and Thy1.2 Pac Blue antibodies.

### Isolation of T cells and proliferation analyses

T cells were isolated from indicated tissues as previously described [6, 7]. Donor cells were distinguished by Thy1.1 and Thy1.2 mAbs. For proliferation analysis using carboxyfluorescein succinimidyl ester (CFSE), naïve P14 CD8 T cells were labeled with CFSE (Molecular Probes, Eugene, OR) and 10^6^ antigen-specific cells were adoptively transferred into naïve mice ~12 hours prior to infection with LCMV. For *in vitro* T cell stimulation, CFSE-labeled wild-type or *dicer*^*CKO*^ P14 cells were plated at 4x10^5^ cells per well in a 96-well flat-bottom plate and stimulated with plate-bound antibodies, anti-CD28 antibody (clone PV-1) (5 μg/mL) and anti-CD3ε (Clone 145-2C11) (5 μg/mL). Alternatively, stimulation was achieved using soluble GP33 peptide (0.27 μg/mL) and anti-CD28 antibody (clone PV-1) (5 μg/mL). Proliferation analysis platform in FlowJo (Treestar) was used to analyze cell division. *In vitro* BrdU (Sigma-Aldrich, St. Louis, MO) was administered at 3.1 μg/well for a period of two hours before the end of the stimulation period, and proliferation was determined by intranuclear staining of BrdU according to BD Biosciences’ (San Jose, CA) protocol.

### Gene deletion analysis

Donor Thy1.1+ CD8 T cells were purified by magnetic bead purification from naïve mice and mice infected with LCMV 7 days prior. Negative CD8 T cell isolation kit was used in conjunction with Thy1.2 positive isolation kit to remove Thy1.2+ CD8 T cells (Stem Cell Technologies, Vancouver, BC, Canada). Purified CD8 T cells were resuspended in 250μL lysis buffer (50mM Tris, 2.5mM EDTA, 50mM KCl, 0.45% NP40, 0.45% Tween-20, pH 8) supplemented with 4 units Proteinase K (New England Biolabs, Ipswich, MA) and incubated at 55°C for 12h. PCR was then performed using the following primers for screening *dicer* deletion [20]: For: 5’-CCT GAC AGT GAC GGT CCA AAG-3’ Rev: 5’- CAT GAC TCT TCA ACT CAA ACT-3’. Primers for *gzmb-cre* PCR were as follows: Tg1: 5’- GCG GTC TGG CAG TAA AAA CTA TC-3’, Tg2: 5’-GTG AAA CAG CAT TGC TGT CAC TT-3’, IPC For: 5’-CTA GGC CAC AGA ATT GAA AGA TCT-3’, IPC Rev: 5’-GTA GGT GGA AAT TCT AGC ATC ATC C-3’. PCR products were then analyzed by agarose gel electrophoresis (1.5%).

For the detection of Dicer function, RT-PCR analysis of miR18a was used. MiRNAs were isolated from the equal numbers of purified WT P14 and *dicer*^*fl/fl*^ *gzmb-cre* P14 CD8 T cells using the miRNeasy Kit (Qiagen, Germantown, MD). Equal amount of RNA was then used to generate cDNA, followed by RT-PCR using miScript primer assays for miR18a and the RNU6-2 control supplied in the miScript PCR Starter Kit (Qiagen, Germantown, MD). ΔΔCt method was used and miR18a expression is shown as fold increase over naïve samples.

### Statistical analysis

Paired or unpaired Student’s t-test was used as indicated to evaluate the differences between sample means of two groups. Mixed ANOVA was performed when analyzing effects of *dicer* ablation over time. When a statistically significant interaction occurred, the difference between groups at each time-point (simple main effect for group) was determined using a general linear model univariate analysis with a Tukey post-hoc test. Mixed ANOVA was performed using IBM SPSS 22. All other statistical analyses were performed using Graphpad Prism 5 and P values of statistical significance are depicted by asterisk per the Michelin guide scale: * (P ≤ 0.05), ** (P ≤ 0.01), *** (P ≤ 0.001) and (P > 0.05) was considered not significant (ns).

## Results

### Diminished CD8 T cell expansion in the absence of Dicer

MicroRNAs have been shown to exert a critical role in CD8 T cell activation and proliferation [13, 14]. Expression of miRNAs in naïve cells is proposed to largely regulate activation, effector differentiation, and proliferation during priming. To specifically query the functional requirements of miRNAs during post-priming stages, we employed the strategy of conditionally deleting *dicer* [20] in mature peripheral T cells after initial priming and activation. Since transcriptional activity of the granzyme B (*gzmb*) promoter is rapidly upregulated in all antigen-specific T cells following activation, we created transgenic *gzmb-cre dicer*^*fl/fl*^ mice (*dicer*^*CKO*^) in which the *gzmb* promoter-drives expression of Cre recombinase. Our approach of conditionally deleting *dicer* in mature peripheral T cells that had upregulated the expression of GzmB was predicted to bypass Dicer requirements during thymic development [21] and priming [13, 14]. Consistent with this, naïve, uninfected mice exhibited normal thymic development of CD4 and CD8 T cells, and ratios of mature CD4 and CD8 T cells in the periphery were similar to wild-type (WT) mice (data not shown).

**Fig 1:**
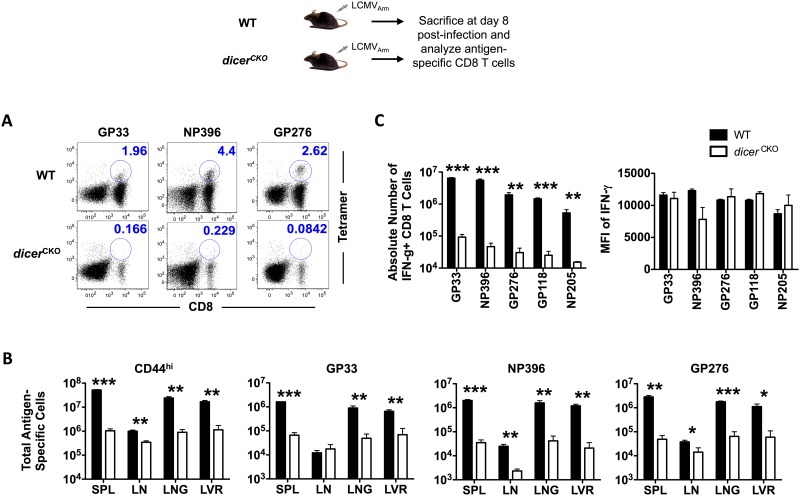
Defective CD8 T cell expansion in the absence of Dicer. C57BL/6 and *dicer*^*CKO*^ mice were infected with LCMV and sacrificed on day 8 post-infection. **(A)** FACS plots show representative data of splenocytes stained for D^b^GP33-, D^b^NP396-, and D^b^GP276-specific CD8 T cells. CD8 T cell frequencies were obtained using MHC-I tetramer staining. Frequencies show percent of total splenocytes. **(B)** Bar graphs depict total numbers of antigen-specific CD8 T cells in spleen, inguinal lymph nodes, lung, and liver. **(C)** Antigen-specific CD8 T cells were analyzed via 5 hour direct *ex vivo* peptide re-stimulation. IFN-γ production was analyzed using intracellular cytokine staining. Bar graphs depict number and MFI of IFN-γ of IFN-γ+ CD8 T cells in spleen. Bar graphs display mean and SEM. Unpaired Student’s t-test was used with statistical significance in difference of means represented as * (P ≤ 0.05), ** (P ≤ 0.01), *** (P ≤ 0.001). Experiments are representative of 2 experiments with 3 mice per group.

We then analyzed antigen-specific CD8 T cell responses in *dicer*^*CKO*^ mice following infection with LCMV. We observed significantly lower numbers of *dicer*^*CKO*^ CD8 T cells compared to WT cells at the peak of effector expansion—three distinct LCMV epitope-specific CD8 T cells (GP33, NP396, and GP276) were markedly reduced following ablation of Dicer (Fig 1A). Consistent with decreased frequencies of tetramer+ CD8 T cells, absolute numbers of antigen-specific D^b^GP33+, D^b^GP276+, and D^b^NP396+ CD8 T cells were diminished by 10–20 fold in most lymphoid and non-lymphoid organs analyzed (Fig 1B). Likewise, the composite LCMV-specific pool of effector cells, as identified by CD44^Hi^ expression [22], was also reduced in the absence of microRNAs post-activation. At memory time-points also, *dicer*^*CKO*^ CD8 T cells exhibited similar reduction in absolute numbers in most lymphoid and non-lymphoid tissues analyzed (S1A and S1B Fig). Expression levels of granzyme B remained similar in WT and *dcr*^*CKO*^ CD8 T cells at the peak of CD8 T cell expansion (S1C Fig). *In vitro* stimulation of CD8 T cells was also associated with similar upregulation of GzmB expression (S1C Fig) in *dcr*^*CKO*^ and WT cells. These data indicate that the conditional deletion system does not alter the levels or kinetics of expression of the effector molecule GzmB. Robust effector differentiation in *dicer*^*CKO*^ antigen-specific CD8 T cells was further supported by similar amounts of effector molecule IFN-γ being produced following *in vitro* stimulation with cognate peptide antigen (Fig 1C). These data indicate that miRNAs exert an important effect on the magnitude of antigen-specific effector CD8 T cell responses, without evident impact on effector differentiation during the post-activation stages.

To confirm *dicer* deletion in CD8 T cells, we performed a PCR for the floxed 5’ site [20] in purified CD8 T cells (90% purity) at days 0 and 7 post-LCMV infection time points (S1D Fig). Only naïve *dicer*^*CKO*^ CD8 T cells showed a band (420bp) for the floxed *dicer* allele and purified *dicer*^*CKO*^ P14 effector CD8 T cells on day 7 showed no such band, confirming effective deletion of Dicer in all *dicer*^*CKO*^ effector cells. The only band appearing in the *dicer*^*CKO*^ sample was the WT band (351bp), possibly stemming from the 10% impurity of WT cells after the magnetic purification. A *gzmb-cre* PCR of the naïve samples confirmed the presence of *cre* in the *dicer*^*CKO*^ mouse (S1E Fig).

Deletion of *dicer* has been shown to delete most prototypical miRNAs [16][15, 23] that are almost exclusively processed by Dicer [16][24][25]. To confirm loss of functional Dicer in CD8 T cells post-activation, we used RT-PCR analysis to measure the quantities of miR18a. Encoded within the miR17-92 cluster, we have previously shown that miR18a is strongly upregulated in CD8 T cells upon stimulation [8]. Whereas stimulated WT CD8 T cells showed continued upregulation of miR18a, *dicer*^*CKO*^ CD8 T cells did not exhibit significant accumulation of miR18a between days 1.5–2.5 after activation (S1F Fig). Thus, RISC functionality was impaired as early as 1.5 days post-activation. These data are consistent with rapid upregulation of GzmB within 24 hours after activation (data not shown) and with previously reported half-life for Dicer protein of about 12–16 hours [26].

### Dicer functions in a CD8 T cell-intrinsic manner to drive CD8 T cell expansion

**Fig 2:**
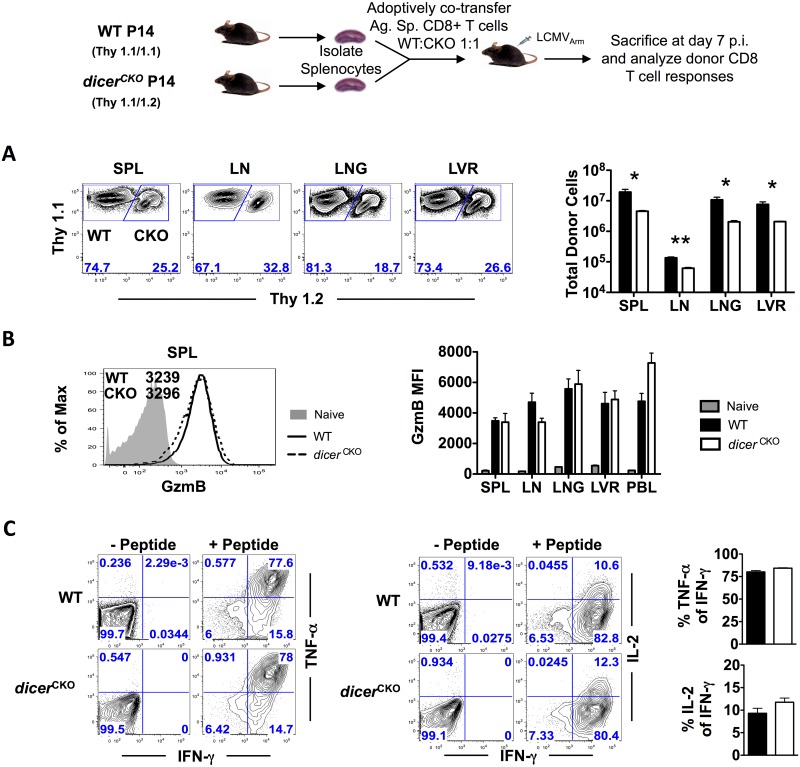
Decreased numbers of *dicer*^*CKO*^ P14 effector cells demonstrate an intrinsic role of Dicer during CD8 T cell expansion. P14 chimeric mice containing 5x10^4^ D^b^GP33-specific WT P14 as well as *dicer*^*CKO*^ P14 CD8 T cells were infected with LCMV and sacrificed at day 7 post-infection. **(A)** FACS plots depict WT and *dicer*^*CKO*^ frequencies of donor antigen-specific CD8 T cells. Bar graphs show total numbers of WT and *dicer*^*CKO*^ donor antigen-specific CD8 T cells in spleen, inguinal lymph nodes, lung, and liver. **(B)** Histogram shows GzmB expression in naïve (grey), WT (black line), and *dicer*^*CKO*^ (dashed line) CD8 T cells on day 7 post-infection in spleen. Bar graphs shows GzmB mean fluorescence intensity (MFI) in spleen, lymph node, lung, liver, and blood. **(C)** Antigen-specific CD8 T cells were analyzed via 5 hour direct *ex vivo* peptide re-stimulation. IFN-γ, TNF-α, and IL-2 production was analyzed using intracellular cytokine staining. FACS plots show donor WT or *dicer*^*CKO*^ IFN-γ+ CD8 T cells. Bar graphs depict percent of TNF-α+ or IL-2+ of IFN-γ+ CD8 T cells of WT or *dicer*^*CKO*^ donor antigen-specific T cells. Bar graphs display mean and SEM. Paired Student’s t-test was used with statistical significance in difference of means represented as * (P ≤ 0.05), ** (P ≤ 0.01), *** (P ≤ 0.001). Experiments are representative of 2 experiments with 3 mice per group.

In the initial experiments, we used straight *dicer*^*CKO*^ mice. However, since NK cells and CD4 T cells also express granzyme B [27] after activation, it is possible that defective CD8 T cell expansion observed in these mice could result indirectly from defective CD4 T cells [1, 3] or NK cells [28, 29] lacking Dicer post-activation. Additionally, due to defective NK, CD4 T cell, and CD8 T cell responses, pathogen clearance is also compromised in these mice (data not shown and [10]). Thus, we next sought to investigate the CD8 T cell-intrinsic requirement of Dicer. For this, we adoptively co-transferred congenically mismatched, TCR transgenic WT and *dicer*^*CKO*^ P14 CD8 T cells in a 1:1 ratio into C57BL/6 recipients. This experimental setup ensures normal viral clearance and Dicer/miRNA deletion only in a subset of antigen-specific CD8 T cells. Despite similar proportions prior to infection, deletion of Dicer/miRNAs after infection and activation led to significantly reduced proportions and absolute numbers of D^b^GP33-specific CD8 T cells in all lymphoid and non-lymphoid organs analyzed (Fig 2A); *dicer*^*CKO*^ donor CD8 T cells were about 5-fold lower in spleen, 2-fold lower in inguinal lymph nodes, and 5-3-fold lower in liver and lungs compared to their WT counterparts (Fig 2A). Consistent with the straight infection data (S1C Fig), GzmB expression was unaltered in the absence of *dicer* (Fig 2B), suggesting that miRNAs are dispensable for regulating effector differentiation during post-activation stages of CD8 T cell responses. To further confirm this notion, we compared the functional capabilities of WT and Dicer^-/-^ effector CD8 T cells to co-produce effector cytokines such as IFN-γ and TNF-α along with common γ-chain cytokine IL-2, known to drive effector differentiation. We observed no differences between WT and *dicer*^*CKO*^ CD8 T cells in their ability to produce IFN-γ, TNF-α, and IL-2 (Fig 2C). These data demonstrate that while miRNAs do not regulate effector molecule expression during post-activation stages, they exert a critical CD8 T cell-intrinsic role in promoting expansion of effector CD8 T cells following optimal activation.

### MicroRNAs promote sustained proliferation for robust CD8 T cell expansion

**Fig 3:**
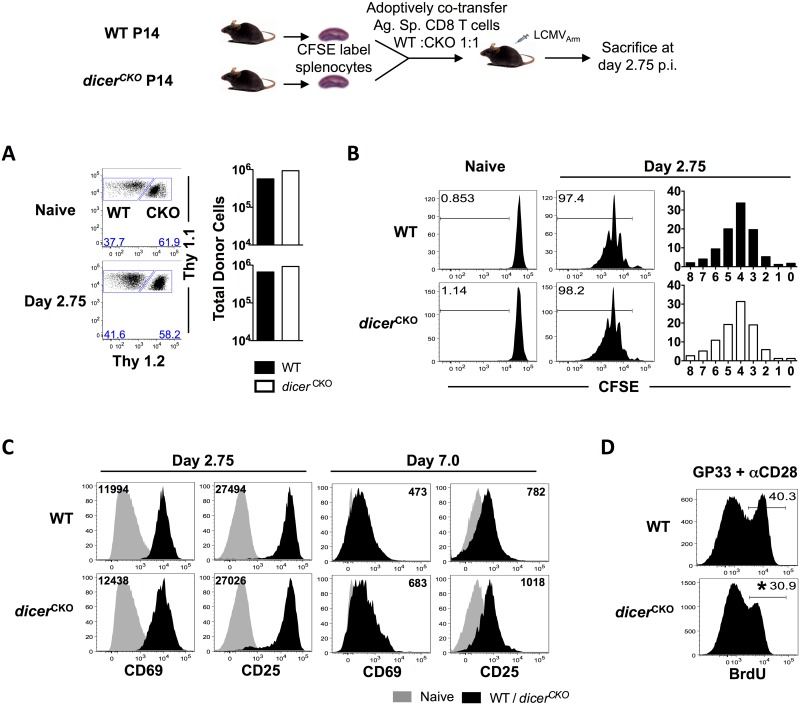
Compromised antigen-driven proliferation upon ablation of Dicer following activation and effector differentiation. WT and *dicer*^*CKO*^ CD8 T cells were labeled with CFSE and adoptively transferred into C57BL/6 recipients. Mice were infected with LCMV and sacrificed 2.75 days later. **(A)** FACS plots show WT and *dicer*^*CKO*^ donor CD8 T cells. Bar graphs show the total number of WT and *dicer*^*CKO*^ donor CD8 T cells. **(B)** Histograms show cell proliferation indicated by CFSE dilution. Bar graphs show frequency of cells per round of cell division at day 2.75 post-infection. **(C)** Representative histogram plots show WT and *dicer*^*CKO*^ donor CD8 T cells from P14 chimeras on day 2.75 and day 7 post-infection. Black numbers in histograms represent MFI. Grey histograms represent a naïve control. **(D)** WT and *dicer*^*CKO*^ P14 splenocytes were stimulated with GP33 and αCD28 *in vitro* for 2.5 days. BrdU was administered 2h prior to the end of the incubation period. Numbers in histograms show percent of BrdU incorporating cells. Unpaired Student’s t-test was used with statistical significance in difference of means represented as * (P ≤ 0.05), ** (P ≤ 0.01), *** (P ≤ 0.001). Experiments are representative of 2 experiments with 3 mice per group.

Decreased numbers of *dicer*^*CKO*^ effector CD8 T cells at the peak of expansion could result from either decreased proliferation or reduced survival of donor cells due to loss of miRNAs. Thus, we next measured the extent and rate of proliferation of WT and *dicer*^*CKO*^ donor CD8 T cells using the cell permeant fluorescent dye CFSE, which gets equally divided between daughter cells with each cell division. We adoptively co-transferred CFSE-labeled D^b^GP33-specific WT and *dicer*^*CKO*^ cells into WT recipients and analyzed their proliferation 2.75 days after LCMV infection. At this early stage, shortly after priming when *dicer* deletion was initiated, both WT and *dicer*^*CKO*^ CD8 T cells were present in equal numbers in the donor mice (Fig 3A). Moreover, WT and *dicer*^*CKO*^ CD8 T cells proliferated equally with the same frequency of cells detectable in each round of cell division 2.75 days post LCMV-infection (Fig 3B). These observations were further supported by similar proliferation after *in vitro* TCR stimulation (S2A Fig), as well as similar upregulation of markers of early activation (CD69 and the IL-2Rα chain, CD25) on WT and *dicer*^*CKO*^ CD8 T cells (Fig 3C). On day 7 post-infection, CD69 and CD25 were expressed at marginally higher levels on *dicer*^*CKO*^ CD8 T cells compared to their WT counterparts in spleen as well as other tissues such as blood (CD25), lymph node and liver (CD69) (Fig 3C and S2B Fig), suggesting that miRNAs may be involved in the downregulation of these markers during later stages of infection. The slightly increased expression of CD69, however, did not impair migration of CD8 T cells into the periphery as *dicer*^*CKO*^ CD8 T cells were present in spleen, lymph node, lung, and liver at similar ratios as WT CD8 T cells (Fig 2A). Despite similar proliferation during early stages after activation, reduced BrdU incorporation by *dicer*^*CKO*^ CD8 T cells during later time-points (58–60 hr window post-stimulation) (Fig 3D) indicates that sustenance of antigen-driven proliferation is dependent on the presence of miRNAs during later stages, when antigen is limiting. Consistent with this notion, *dicer*^*CKO*^ CD8 T cells were less responsive to TCR stimulation compared to WT CD8 T cells (S2C Fig). At lower concentrations of stimulating peptide, lesser BrdU incorporation was observed in *dicer*^*CKO*^ CD8 T cells compared to WT cells. To explore the possibility of increased death in *dicer*^*CKO*^ CD8 T cells, we performed a caspase assay after *in vitro* stimulation (S2D Fig). Both WT and *dicer*^*CKO*^ CD8 T cells expressed the same amount of caspases 3 and 7 post-activation suggesting that *dicer*^*CKO*^ CD8 T cells do not exhibit increased cell death during the effector phase. These data illustrate that WT and *dicer*^*CKO*^ CD8 T cells are activated equally and proliferate identically early after LCMV infection. In the later stages of the effector response, however, the dominantly pro-proliferative role of miRNAs becomes apparent, as proliferation is impaired upon *dicer* deletion, which ultimately results in reduced total numbers of antigen-specific CD8 T cells at the peak of expansion.

### MicroRNAs promote survival of antigen-specific CD8 T cells during contraction

**Fig 4:**
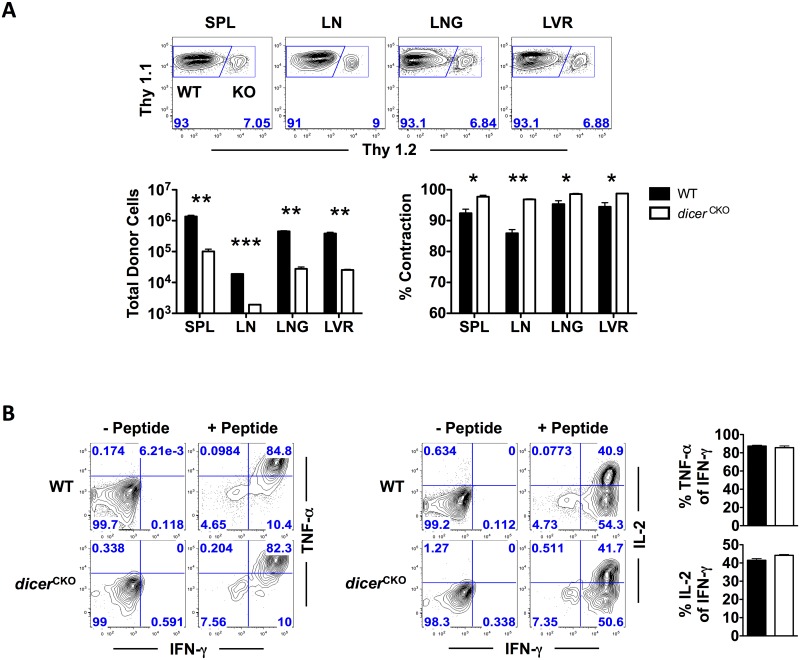
Ablation of Dicer in CD8 T cells results in reduced memory CD8 T cell numbers. P14 chimeric mice containing 5x10^4^ D^b^GP33-specific WT P14 as well as *dicer*^*CKO*^ P14 CD8 T cells were infected with LCMV and sacrificed at memory (D44). **(A)** FACS plots depict WT and *dicer*^*CKO*^ frequencies of donor antigen-specific CD8 T cells. Bar graphs show total numbers as well as percent contraction between effector and memory time points of WT and *dicer*^*CKO*^ donor antigen-specific CD8 T cells in spleen, inguinal lymph nodes, lung, and liver. **(B)** Antigen-specific CD8 T cells were analyzed via direct *ex vivo* re-stimulation as described before and production of IFN-γ, TNF-α, and IL-2 is presented. FACS plots show donor WT or *dicer*^*CKO*^ IFN-γ+ CD8 T cells. Bar graphs depict percent of TNF-α+ or IL-2+ of IFN-γ+ CD8 T cells of WT or *dicer*^*CKO*^ donor antigen-specific T cells. Bar graphs display mean and SEM. Paired Student’s t-test was used with statistical significance in difference of means represented as * (P ≤ 0.05), ** (P ≤ 0.01), *** (P ≤ 0.001). Experiments are representative of 2 experiments with 3 mice per group.

About 90–95% of effector CD8 T cells present at the peak of expansion are short-lived and die through apoptotic elimination in the contraction phase whereas 5–10% of the remaining cells downregulate their effector program and differentiate into quiescent memory cells [7]. To determine whether, in addition to regulating CD8 T cell expansion, miRNAs also exert an impact on contraction and memory formation, we enumerated the numbers of WT and *dicer*^*CKO*^ antigen-specific CD8 T cells at memory and calculated the extent of contraction in both subsets from peak to memory. At memory, we observed more than 10-fold lower numbers of *dicer*^*CKO*^ CD8 T cells compared to WT cells in both lymphoid as well as non-lymphoid organs, albeit *dicer*^*CKO*^ CD8 T cells showed no difference in localization in lymphoid and non-lymphoid tissues (Fig 4A). Further reduced numbers of *dicer*^*CKO*^ CD8 T cells at memory compared to day 7 after infection suggested enhanced contraction in the absence of miRNAs. This prediction was validated by greater contraction/death of antigen-specific *dicer*^*CKO*^ CD8 T cells in all organs relative to WT donor cells (Fig 4A). An assessment of the polyfunctionality of WT and *dicer*^*CKO*^ donor CD8 T cells revealed similar functional potency (Fig 4B); WT and *dicer*^*CKO*^ CD8 T cells co-produced similar levels of IFN-γ, TNF-α, and IL-2 on a per cell basis. Memory *dicer*^*CKO*^ CD8 T cells, however, showed modestly higher expression of GzmB, CD69, CD25, and PD-1, likely due to defects in their turnover in the absence of miRNAs (S3 Fig). Together, these data indicate that Dicer/miRNA expression during post-activation stages is not needed for robust polyfunctionality or disperse localization of memory CD8 T cells in lymphoid and nonlymphoid tissues. However, Dicer-dependent miRNAs promote quantitative properties of antigen-specific CD8 T cell memory elicited in response to acute viral infection.

### MicroRNAs regulate MPEC and SLEC lineages

**Fig 5:**
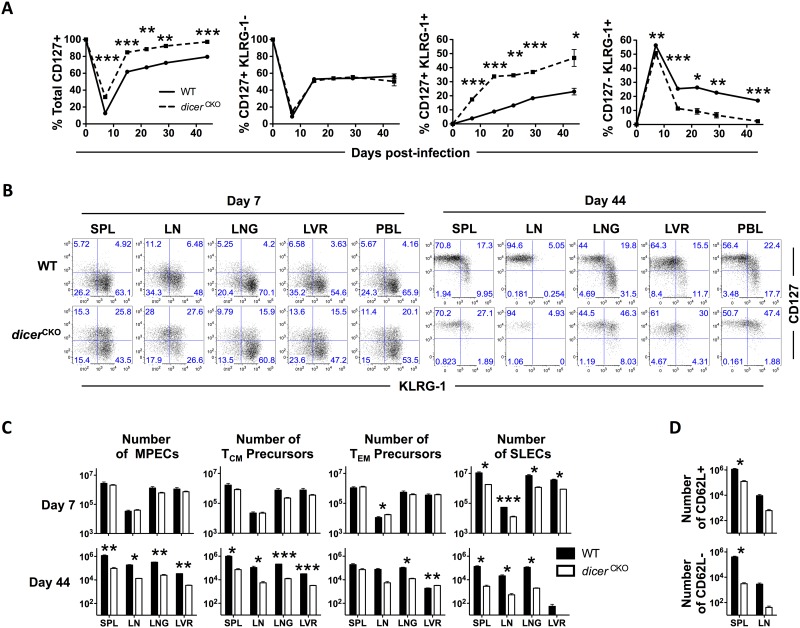
Characterization of effector and memory lineage differentiation of *dicer*^*CKO*^ antigen-specific CD8 T cells. P14 chimeric mice containing 5x10^4^ D^b^GP33-specific WT P14 as well as *dicer*^*CKO*^ P14 CD8 T cells were infected with LCMV and sacrificed at the peak of effector expansion and memory. **(A)** Line graphs show the longitudinal frequency of WT and *dicer*^*CKO*^ CD8 T cell subsets in blood based on their expression of CD127 and KLRG-1. **(B)** FACS plots show CD127 and KLRG-1 expression of WT and *dicer*^*CKO*^ donor CD8 T cells on day 7 and 44 post-LCMV infection in spleen, lymph node, lung, liver, and blood. **(C)** Bar graphs show numbers of MPEC (CD127+), T_CM_ (CD127+KLRG-1-), T_EM_ (CD127+KLRG-1+), SLEC (CD127-KLRG-1+) donor WT and *dicer*^*CKO*^ antigen-specific CD8 T cells at day 7 and day 44 post-infection in spleen, inguinal lymph nodes, lung, and liver. **(D)** Bar graphs show numbers of CD62L+ and CD62L- donor WT and *dicer*^*CKO*^ antigen-specific CD8 T cells at day 70 post-infection in spleen and inguinal lymph nodes. Line and bar graph display mean and SEM. Mixed ANOVA was used to compare longitudinal PBMC data (A). Otherwise paired Student’s t-test was used. Statistical significance in difference of means is represented as * (P ≤ 0.05), ** (P ≤ 0.01), *** (P ≤ 0.001). Experiments are representative of 2 experiments with 3 mice per group.

Based on increased contraction of *dicer*^*CKO*^ CD8 T cells and a diminished memory compartment, we next investigated whether Dicer/miRNAs regulate the commitment of antigen-specific CD8 T cells to the memory and the terminal effector lineages during the expansion phase following optimal activation. Memory-fated MPEC and the short-lived SLEC lineages are distinguishable during the CD8 T cell expansion phase by differential expression of cell surface markers IL-7Rα [30, 31] and KLRG-1 [7, 32]. MPECs selectively express higher levels of IL-7Rα (CD127), whereas SLECs are distinguished by a CD127^Lo^KLRG-1^Hi^ phenotype. A further distinction of the long-lived, polyfunctional, lymphoid-homing CD62L+ central memory (T_CM_) lineage and the recirculating, nonlymphoid-homing CD62L- effector memory (T_EM_) lineage is marked by higher expression of KLRG-1 and CD127 on T_EM_ cells [32][7]. To our surprise, we found that despite lower numbers of antigen-specific CD8 T cells at memory, *dicer*^*CKO*^ CD8 T cells were enriched in CD127^Hi^ MPECs at the effector peak as well as in memory in lymphoid and non-lymphoid tissues (Fig 5A and 5B) compared to WT CD8 T cells. Additionally, *dicer*^*CKO*^ CD8 T cells contained relatively lower proportions of CD127^Lo^KLRG-1^Hi^ SLECs compared to WT donor counterparts (Fig 5A). Over time, *dicer*^*CKO*^ remained higher for CD127 expression than WT donor cells (Fig 5A). Within the MPEC pool of CD127^Hi^ cells, we found that the CD127^Hi^KLRG-1^Lo^ subset that primarily gives rise to T_CM_ cells was similar, whereas the CD127^Hi^KLRG-1^Hi^ subset that largely differentiates into T_EM_ cells was increased in *dicer*^*CKO*^ CD8 T cells (Fig 5A). Conversely, CD127^Lo^KLRG-1^Hi^ SLECs were reduced in *dicer*^*CKO*^ CD8 T cells at the peak of expansion and decreased more rapidly after viral clearance than their WT counterparts (Fig 5A). Notably, changes in the balance between MPECs and SLECs did not correlate with alterations in the expression of the pro-survival molecule Bcl-2. Bcl-2 was expressed at higher levels in *dicer*^*CKO*^ CD8 T cells during both the effector and memory phases (S4 Fig). Together, these findings show that miRNAs regulate the balance of terminal effector and memory fates during the exponential expansion of CD8 T cells.

To gain a better understanding of Dicer/miRNA-dependent regulation of MPEC and SLEC fates, we compared absolute numbers of total MPEC (CD127^Hi^), SLEC (CD127^Lo^), T_CM_ precursor (CD127^Hi^KLRG-1^Lo^) and T_EM_ precursor (CD127^Hi^KLRG-1^Hi^) WT and *dicer*^*CKO*^ antigen-specific CD8 T cells at the peak of expansion. The SLEC subset was evidently reduced in *dicer*^*CKO*^ antigen-specific CD8 T cells compared to WT cells at the peak (Fig 5C). In contrast, peak MPEC, T_EM_ and T_CM_ precursor cell numbers were largely unaffected upon Dicer ablation (Fig 5C). Notably, following contraction, all subsets (MPEC, SLEC, T_EM_ precursors and T_CM_ precursors) were lower in the *dicer*^*CKO*^ antigen-specific CD8 T cells (Fig 5C). Consistent with this, both CD62L- T_EM_ and CD62L+ T_CM_ numbers were reduced in *dicer*^*CKO*^ antigen-specific CD8 T cells compared to WT cells at memory (Fig 5D). These observations demonstrate that Dicer/miRNAs preferentially drive the expansion of SLECs during the CD8 T cell expansion phase, and promote prolonged survival of memory-fated as well as short-lived antigen-specific CD8 T cells during contraction and memory phases.

## Discussion

As the variety of cell types and functions known to be regulated by miRNAs is continuously expanding, there is increasing interest in understanding how microRNAs regulate immune responses [11, 12, 33–35]. In this study, we investigated the effect of Dicer/miRNAs on CD8 T cell effector and memory differentiation by selectively inactivating Dicer after CD8 T cell activation. Our results demonstrate a critical requirement of miRNAs in promoting the magnitude of effector CD8 T cells and regulating the balance of terminal effector and memory lineages by driving continued proliferation of terminal effector cells and controlling the contraction of memory-fated as well as short-lived effector cells. By focusing on post-activation stages, these studies not only highlight a novel and distinct role of miRNAs in effector and memory development, which was missed thus far due to severe defects in CD8 T cell activation when *dicer* was deleted prior to activation, but also provide novel insight into the potential physiological processes regulated by miRNAs. These findings in the murine model of acute infection with LCMV, a well-established system for studying fundamental mechanisms of CD8 T cell immunity, bear broad applicability to miRNA regulation of CTL immunity against a variety of acute infections.

Similar to previous studies where miRNAs were ablated prior to T cell priming [13, 14], we observed a diminished peak effector pool upon ablation of Dicer/miRNAs during the post-activation stages. Decreased effector pool was associated with lack of sustained antigen-driven proliferation in the later stages of effector expansion while the functional capabilities of the effector cells remained unperturbed. We found that this decreased expansion in *dicer*^*CKO*^ CD8 T cells was likely due to reduced TCR sensitivity in the absence of mature miRNAs post-activation. Conversely, reduced effector pool size associated with miRNA ablation prior to T cell priming may be attributed to reduced cell survival despite increased proliferation [14]. Our observations are also consistent with previous reports of decreased expansion following deletion of specific pro-proliferative miRNAs such as miR-17~92 and miR-155 [8, 10, 36–39]. We observed modestly increased CD69 expression upon miRNA ablation during post-activation stages, albeit the level of CD69 upregulation was not to the extent reported previously [14] when Dicer was absent from the beginning of CTL activation. Consistent with this, we did not observe alterations in CTL migration associated with *dicer* ablation prior to priming. Our results using the adoptive co-transfer model of *dicer*^*fl/fl*^ P14 and WT P14 CD8 T cells showed drastically lower numbers of memory CD8 T cells that we observed only partially in our straight infections. The lesser difference in memory numbers is likely caused by impaired viral clearance in *dicer*^*fl/fl*^ mice, a confounding factor we fully bypassed in our adoptive transfer setting.

We have previously shown that loss of miR-17~92 post-activation results in decreased expansion and enhanced MPEC proportions [8], similar to our observations with conditional deletion of *dicer* post-activation. However, instead of decreased proportions of T_EM_ CD8 T cells observed upon specific loss of miR-17~92, Dicer/miRNA ablation resulted in an increase in T_EM_ proportions at effector peak. While absolute numbers of T_EM_ and T_CM_ precursors were unaffected at the effector peak, ablation of Dicer/miRNAs led to a pronounced loss of MPECs (both T_EM_ and T_CM_ precursors) and SLECs during contraction and memory phases. In addition to an altered MPEC and SLEC balance, our observations of normal early proliferation following global loss of miRNAs were unlike those when only miR-17~92 was deleted, where we observed impaired proliferation in the early stages of infection. It is important to consider that the miR-17~92 cluster is one amongst many that regulate proliferation either positively or negatively. Thus, several miRNAs may simultaneously exert differential effects on proliferation, survival, and differentiation of CD8 T cells, such that the balance of all relevant miRNA regulatory elements in a cell determines the net physiological effect. Consequently, loss of pro-proliferative miR-17~92 alone may result in decreased proliferation in the early stages of infection due to an altered balance in favor of anti-proliferative miRNAs, whereas deletion of majority of cellular miRNAs has an effect only on sustained proliferation in the later stages leading up to the peak of the response. It is also possible that reduced proliferation is not instantaneously evident in the *dicer*^*CKO*^ CD8 T cells as loss of miRNAs is slightly delayed in *dicer*^*CKO*^ compared to *mir17~92*^*CKO*^ CD8 T cells. While *mir17~92*^*CKO*^ CD8 T cells do not rely on abrogation of RNA-induced silencing complex (RISC) function due to direct deletion of the gene locus following activation of the *gzmb* promoter, residual RISC function in *dicer*^*CKO*^ CD8 T cells is expected to progressively decline after activation of the *gzmb* promoter. Our observations that Dicer-dependent miRNAs fail to progressively increase beyond day 1.5 after infection in *dicer*^*CKO*^ CD8 T cells (once *gzmb* promoter is optimally activated) and previous studies assessing the half-life of Dicer as 12-16hrs [26] support this possibility. Nonetheless, our TCR transgenic model system of *gzmb-cre dicer*^*fl/fl*^ mice provides a foundation for future dissection of the timing of the functional requirement of individual miRNAs in the post-priming stages of effector and memory CTL differentiation by reversal-of-phenotype/function experiments following miRNA transfection.

Collectively, these studies support the proposal [40, 41] that the miRNA repertoire of CD8 T cells is rapidly remodeled following activation, such that the net physiological effect of exponential expansion and optimal effector differentiation is achieved by the simultaneous downregulation of miRNAs inhibiting CD8 T cell expansion and effector differentiation, and upregulation of miRNAs promoting survival and proliferation. Likewise, differential effects of Dicer/miRNA deletion on the balance of central memory, effector memory, and short-lived effector cells further highlight the many facets of miRNA function and the need for tight rheostatic control through multiple miRNAs. In conclusion, these data present a model of dynamic orchestration of miRNA expression and CD8 T cell expansion and differentiation through regulation of key physiological processes.

## Supporting Information

S1 Fig Deletion of Dicer function in CD8 T cells post-activation. **(A-B)** C57BL/6 and *dicer*^*fl/fl*^ *gzmb-cre* mice were infected with LCMV and sacrificed at memory (D>60). **(A)** FACS plots show representative data of splenocytes stained for D^b^GP33-, D^b^NP396-, and D^b^GP276-specific CD8 T cells. CD8 T cell frequencies were obtained using MHC-I tetramer staining. Frequencies show percent of total splenocytes. **(B)** Bar graphs depict total numbers of antigen-specific CD8 T cells in spleen, inguinal lymph nodes, lung, and liver. **(C)** Granzyme B histograms of *dicer*^*fl/fl*^ *gzmb-cre* and C57BL/6 CD8 T cells on day 2.5 post-*in vitro* stimulation with αCD3ε and αCD28 as well as on day 7 post-infection with LCMV in P14 chimeric mice containing 5x10^4^ D^b^GP33-specific WT P14 as well as *dicer*^*fl/fl*^ *gzmb-cre* P14 CD8 T cells. Naïve control is shown as a grey histogram. Numbers in plots represent MFI. **(D)** Gel PCR analysis for presence of *dicer-flox* pre- and post-infection in purified CD8 T cells. For samples from day 7, WT P14 and *dicer*^*fl/fl*^ *gzmb-cre* P14 splenocytes were adoptively transferred into C57BL/6 recipients and infected with LCMV. WT band at 351bp, KO band containing floxed allele at 420bp. **(E)** Gel PCR analysis for presence of *gzmb-cre* pre-infection in purified CD8 T cells. Lanes were rearranged for clarity. WT positive control at 325 bp, KO band showing presence of *gzmb-cre* at 100bp. **(F)** MiR18a expression was quantified as fold-increase over naïve in WT P14 and *dicer*^*fl/fl*^ *gzmb-cre* CD8 T cells. CD8 T cells were magnetically purified to >99% purity and stimulated with GP33 Tetramer and αCD28. RT-PCR was performed with naïve and samples stimulated for 1.5 and 2.5 days. Bar graphs display mean and SEM. Unpaired Student’s t-test was used with statistical significance in difference of means represented as * (P ≤ 0.05), ** (P ≤ 0.01), *** (P ≤ 0.001). Experiments are representative of 2 experiments with 3 mice per group.
(TIFF)

S2 Fig *In vitro* analysis of early expansion and survival of Dicer^-/-^ CD8 T cells. WT P14 as well as *dicer*^*fl/fl*^ *gzmb-cre* P14 CD8 T cells were labeled with CFSE and stimulated with αCD3ε and αCD28 antibodies or GP33 peptide and αCD28. **(A)** CFSE histograms on day 2.5 after *in vitro* stimulation. Numbers show percent of proliferated cells. Grey histograms show naïve control. **(B)** CD25 and CD69 expression of naïve as well as WT and KO donor CD8 T cells on day 7 post LCMV-infection in spleen, lymph node, lung, liver, and blood. Asterisks show statistical significance between WT and KO groups. **(C)** WT and KO splenocytes were stimulated with constant amounts of αCD28 but varying amounts of GP33 peptide. BrdU was administered 2h prior to the end of the incubation period. Numbers in histograms show percent of BrdU incorporating cells. Line graphs show the percent of BrdU+ CD8 T cells for each dilution. **(D)** WT and KO splenocytes were stimulated *in vitro* and presence of caspases 3 and 7 was assessed via the FAM-FLICA Apoptosis Detection Kit from Neuromics. Cells were stained for 20 min at 37°C. Histograms show combined levels of caspases 3 and 7 after 60h of stimulation. Numbers in histograms represent MFI. Bar graphs display mean and SEM. Paired **(B)** or unpaired **(C)** Student’s t-test was used with statistical significance in difference of means represented as * (P ≤ 0.05), ** (P ≤ 0.01), *** (P ≤ 0.001). Experiments are representative of 2 experiments with 3 mice per group.
(TIFF)

S3 Fig Activation marker expression of Dicer^-/-^ CD8 T cells at memory. P14 chimeric mice containing 5x10^4^ D^b^GP33-specific WT P14 as well as *dicer*^*fl/fl*^ *gzmb-cre* P14 CD8 T cells were infected with LCMV and sacrificed at memory. Marker expression of naïve as well as WT and KO donor CD8 T cells on day 70 post LCMV-infection in spleen, lymph node, lung, liver, and blood is shown. Asterisks show statistical significance between WT and KO groups. Bar graphs display mean and SEM. Paired Student’s t-test was used with statistical significance in difference of means represented as * (P ≤ 0.05), ** (P ≤ 0.01), *** (P ≤ 0.001). Experiments are representative of 2 experiments with 3 mice per group.
(TIFF)

S4 Fig Bcl-2 expression in Dicer^-/-^ CD8 T cells at effector and memory stages. P14 chimeric mice containing 5x10^4^ D^b^GP33-specific WT P14 and *dicer*^*fl/fl*^ *gzmb-cre* P14 CD8 T cells were infected with LCMV and sacrificed at the peak of effector expansion and memory. **(A)** Histogram shows Bcl-2 expression of splenocytes in naïve (grey), WT (black line), and *dicer*^*CKO*^ (dashed line) CD8 T cells on day 70 post-infection. Bar graph shows Bcl-2 MFI in spleen at the peak of expansion (D7) and memory stages (D44, D70). **(B)** Bcl-2 expression of naïve as well as WT and KO donor CD8 T cells on day 7 and day 70 post LCMV-infection in spleen, lymph node, lung, liver, and blood is presented. Asterisks show statistical significance between WT and KO groups. Bar graphs display mean and SEM. Paired Student’s t-test was used with statistical significance in difference of means represented as * (P ≤ 0.05), ** (P ≤ 0.01), *** (P ≤ 0.001). Experiments are representative of 2 experiments with 3 mice per group.
(TIFF)
