# Supplementary figures and images for: Dicer Regulates the Balance of Short-Lived Effector and Long-Lived Memory CD8 T Cell Lineages
Source: PLoS One. 2016 Sep 14;11(9):e0162674. doi: 10.1371/journal.pone.0162674 (PMC5023163; doi:10.1371/journal.pone.0162674)

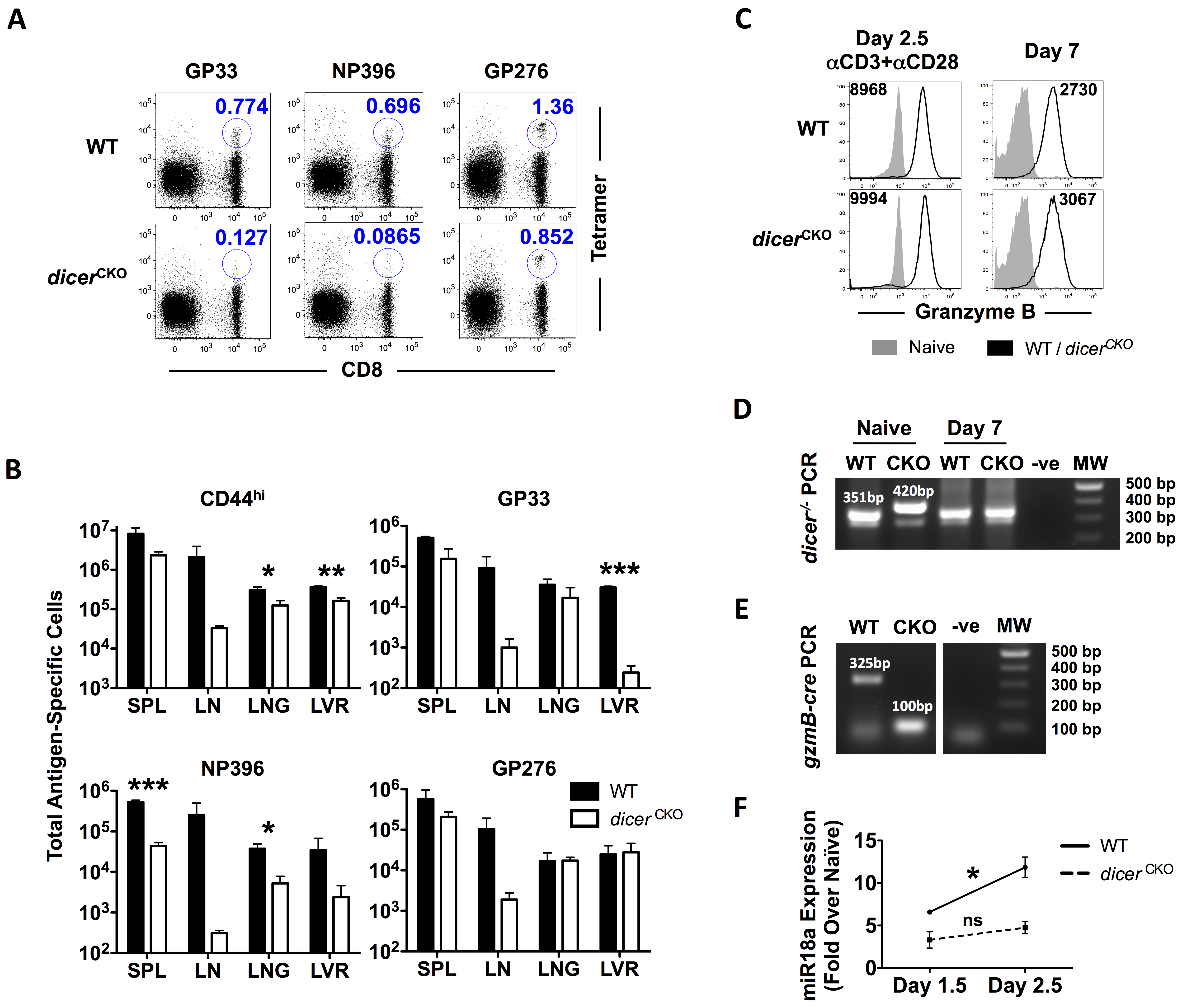

Supplement: S1 Fig — (A-B) C57BL/6 and dicerfl/fl gzmb-cre mice were infected with LCMV and sacrificed at memory (D>60). (A) FACS plots show representative data of splenocytes stained for DbGP33-, DbNP396-, and DbGP276-specific CD8 T cells. CD8 T cell frequencies were obtained using MHC-I tetramer staining. Frequencies show percent of total splenocytes. (B) Bar graphs depict total numbers of antigen-specific CD8 T cells in spleen, inguinal lymph nodes, lung, and liver. (C) Granzyme B histograms of dicerfl/fl gzmb-cre and C57BL/6 CD8 T cells on day 2.5 post-in vitro stimulation with αCD3ε and αCD28 as well as on day 7 post-infection with LCMV in P14 chimeric mice containing 5x10^4^ DbGP33-specific WT P14 as well as dicerfl/fl gzmb-cre P14 CD8 T cells. Naïve control is shown as a grey histogram. Numbers in plots represent MFI. (D) Gel PCR analysis for presence of dicer-flox pre- and post-infection in purified CD8 T cells. For samples from day 7, WT P14 and dicerfl/fl gzmb-cre P14 splenocytes were adoptively transferred into C57BL/6 recipients and infected with LCMV. WT band at 351bp, KO band containing floxed allele at 420bp. (E) Gel PCR analysis for presence of gzmb-cre pre-infection in purified CD8 T cells. Lanes were rearranged for clarity. WT positive control at 325 bp, KO band showing presence of gzmb-cre at 100bp. (F) MiR18a expression was quantified as fold-increase over naïve in WT P14 and dicerfl/fl gzmb-cre CD8 T cells. CD8 T cells were magnetically purified to >99% purity and stimulated with GP33 Tetramer and αCD28. RT-PCR was performed with naïve and samples stimulated for 1.5 and 2.5 days. Bar graphs display mean and SEM. Unpaired Student’s t-test was used with statistical significance in difference of means represented as * (P ≤ 0.05), ** (P ≤ 0.01), *** (P ≤ 0.001). Experiments are representative of 2 experiments with 3 mice per group. (TIFF) [file pone.0162674.s001.tiff]

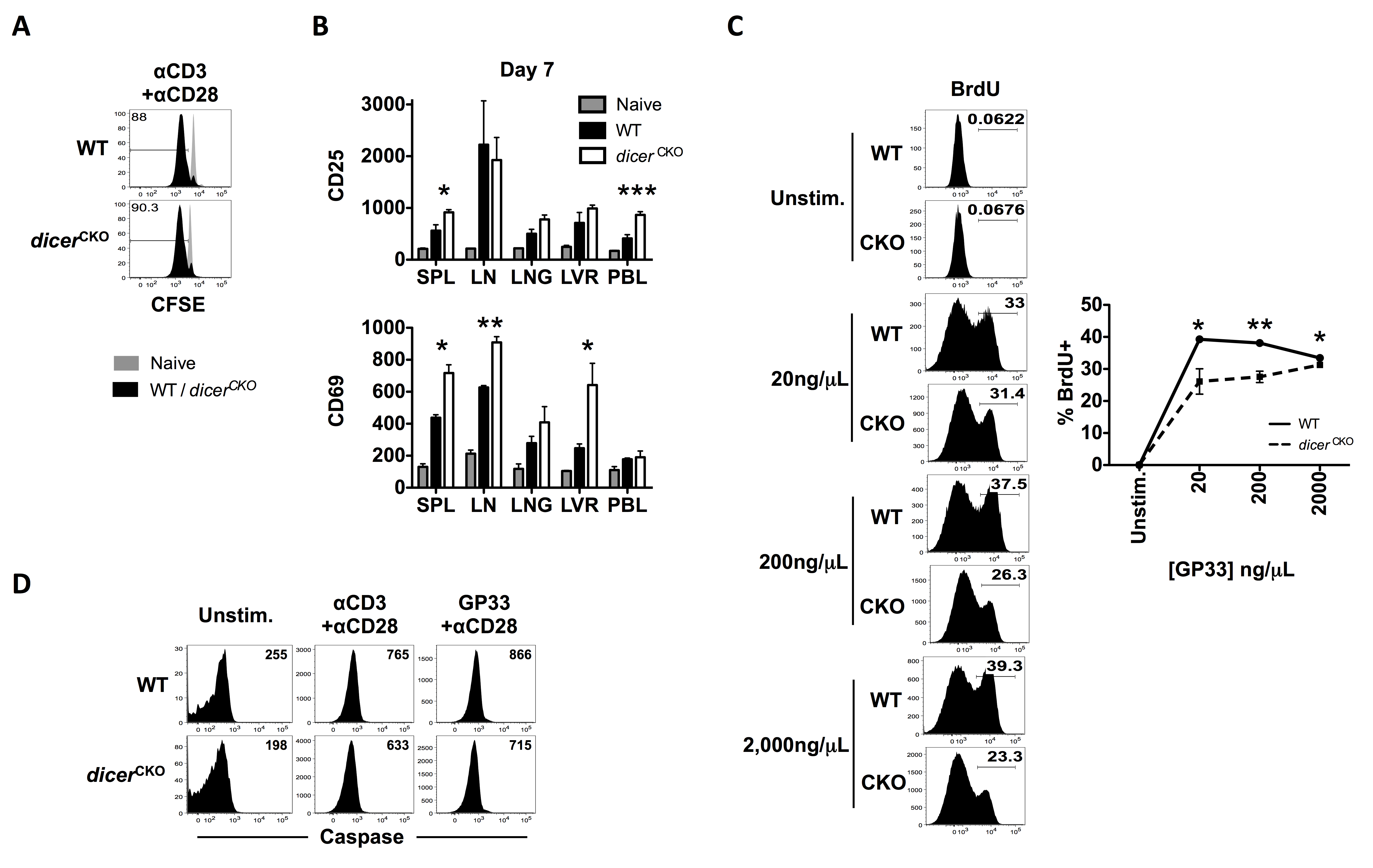

Supplement: S2 Fig — WT P14 as well as dicerfl/fl gzmb-cre P14 CD8 T cells were labeled with CFSE and stimulated with αCD3ε and αCD28 antibodies or GP33 peptide and αCD28. (A) CFSE histograms on day 2.5 after in vitro stimulation. Numbers show percent of proliferated cells. Grey histograms show naïve control. (B) CD25 and CD69 expression of naïve as well as WT and KO donor CD8 T cells on day 7 post LCMV-infection in spleen, lymph node, lung, liver, and blood. Asterisks show statistical significance between WT and KO groups. (C) WT and KO splenocytes were stimulated with constant amounts of αCD28 but varying amounts of GP33 peptide. BrdU was administered 2h prior to the end of the incubation period. Numbers in histograms show percent of BrdU incorporating cells. Line graphs show the percent of BrdU+ CD8 T cells for each dilution. (D) WT and KO splenocytes were stimulated in vitro and presence of caspases 3 and 7 was assessed via the FAM-FLICA Apoptosis Detection Kit from Neuromics. Cells were stained for 20 min at 37°C. Histograms show combined levels of caspases 3 and 7 after 60h of stimulation. Numbers in histograms represent MFI. Bar graphs display mean and SEM. Paired (B) or unpaired (C) Student’s t-test was used with statistical significance in difference of means represented as * (P ≤ 0.05), ** (P ≤ 0.01), *** (P ≤ 0.001). Experiments are representative of 2 experiments with 3 mice per group. (TIFF) [file pone.0162674.s002.tiff]

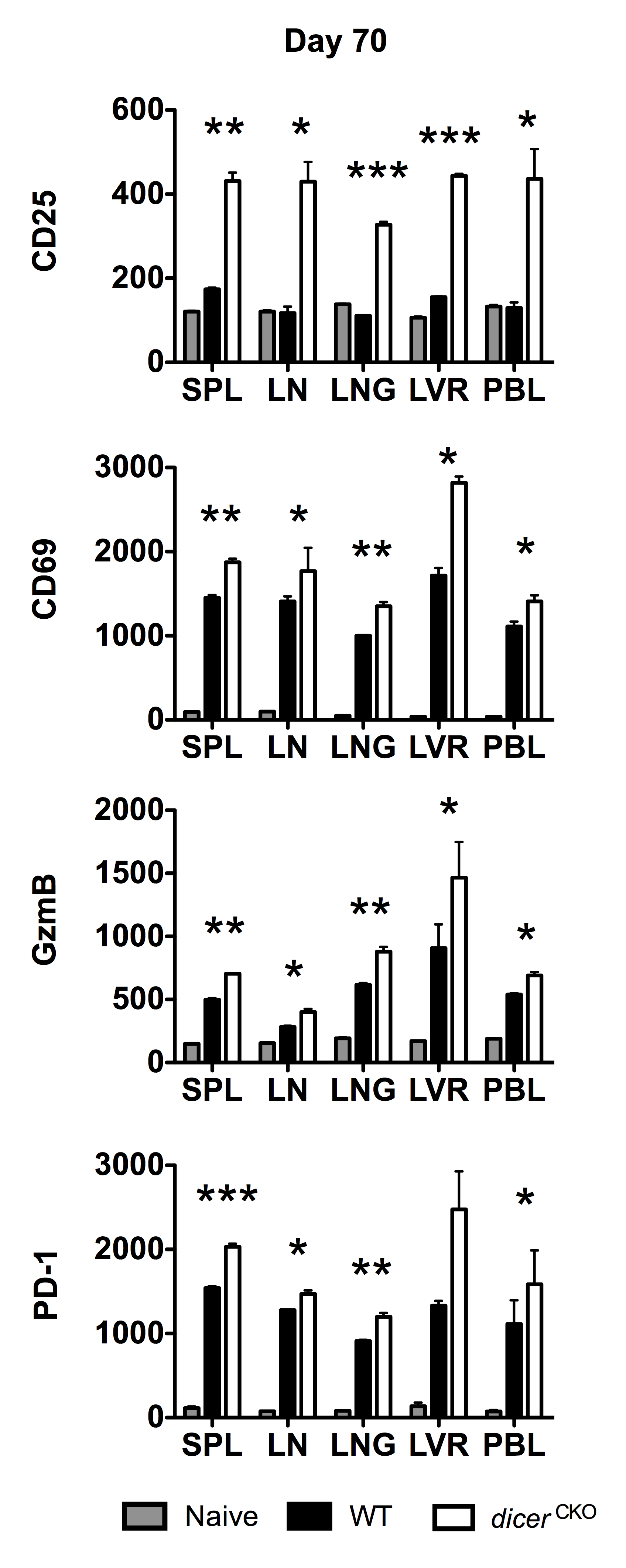

Supplement: S3 Fig — P14 chimeric mice containing 5x10^4^ DbGP33-specific WT P14 as well as dicerfl/fl gzmb-cre P14 CD8 T cells were infected with LCMV and sacrificed at memory. Marker expression of naïve as well as WT and KO donor CD8 T cells on day 70 post LCMV-infection in spleen, lymph node, lung, liver, and blood is shown. Asterisks show statistical significance between WT and KO groups. Bar graphs display mean and SEM. Paired Student’s t-test was used with statistical significance in difference of means represented as * (P ≤ 0.05), ** (P ≤ 0.01), *** (P ≤ 0.001). Experiments are representative of 2 experiments with 3 mice per group. (TIFF) [file pone.0162674.s003.tiff]

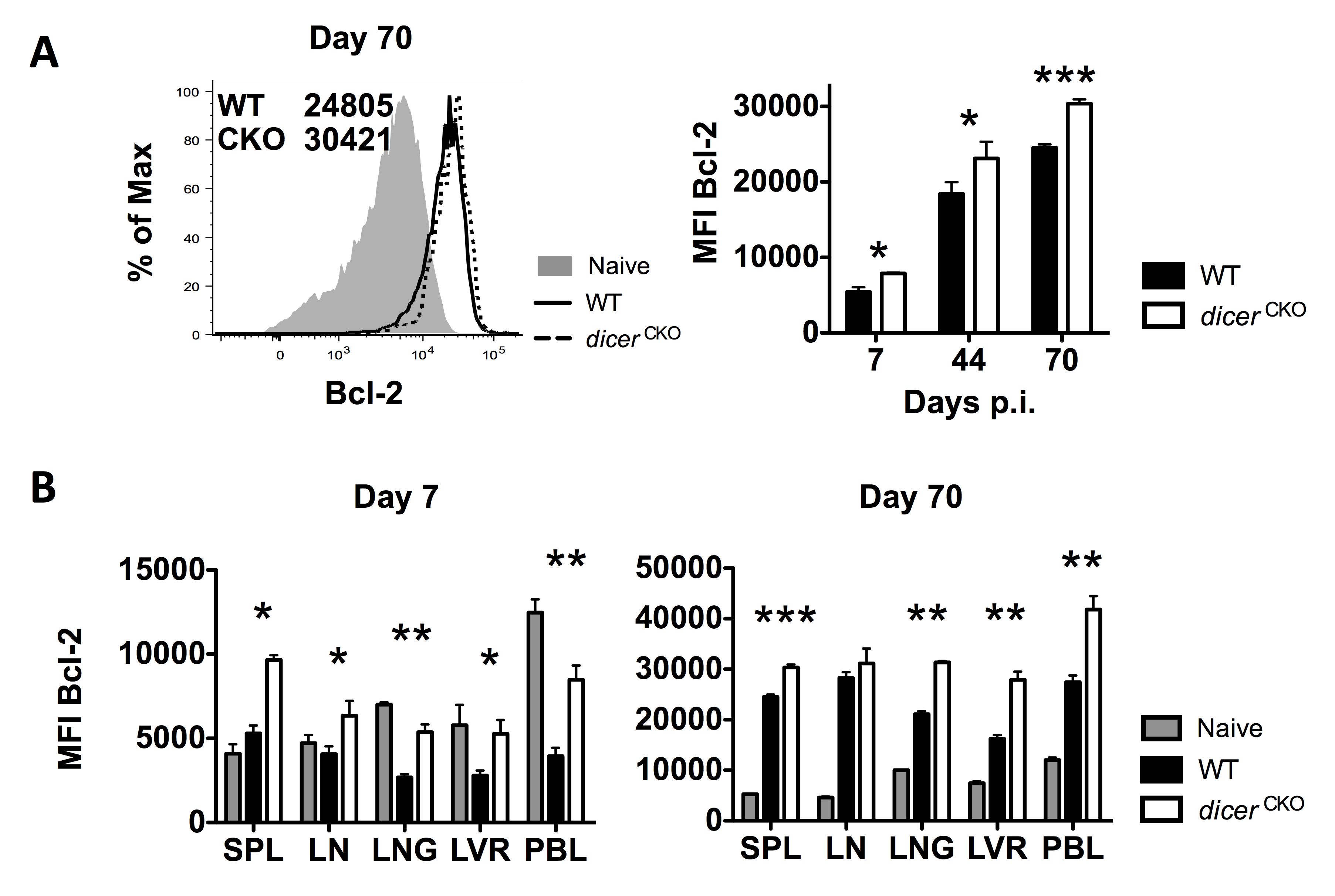

Supplement: S4 Fig — P14 chimeric mice containing 5x10^4^ DbGP33-specific WT P14 and dicerfl/fl gzmb-cre P14 CD8 T cells were infected with LCMV and sacrificed at the peak of effector expansion and memory. (A) Histogram shows Bcl-2 expression of splenocytes in naïve (grey), WT (black line), and dicerCKO (dashed line) CD8 T cells on day 70 post-infection. Bar graph shows Bcl-2 MFI in spleen at the peak of expansion (D7) and memory stages (D44, D70). (B) Bcl-2 expression of naïve as well as WT and KO donor CD8 T cells on day 7 and day 70 post LCMV-infection in spleen, lymph node, lung, liver, and blood is presented. Asterisks show statistical significance between WT and KO groups. Bar graphs display mean and SEM. Paired Student’s t-test was used with statistical significance in difference of means represented as * (P ≤ 0.05), ** (P ≤ 0.01), *** (P ≤ 0.001). Experiments are representative of 2 experiments with 3 mice per group. (TIFF) [file pone.0162674.s004.tiff]
